# Supplementary material for: Association between oral microbiome and five types of respiratory infections: a two-sample Mendelian randomization study in east Asian population
Source: Front Microbiol. 2024 Apr 10;15:1392473. doi: 10.3389/fmicb.2024.1392473 (PMC11039966; doi:10.3389/fmicb.2024.1392473)
Supplement: Supplementary file 1 [file Data_Sheet_1.zip › Supplementary Materials/MR plots of saliva/Pneumonia/s__Streptococcus_mitis_I_mgs_3085/forest.pdf]

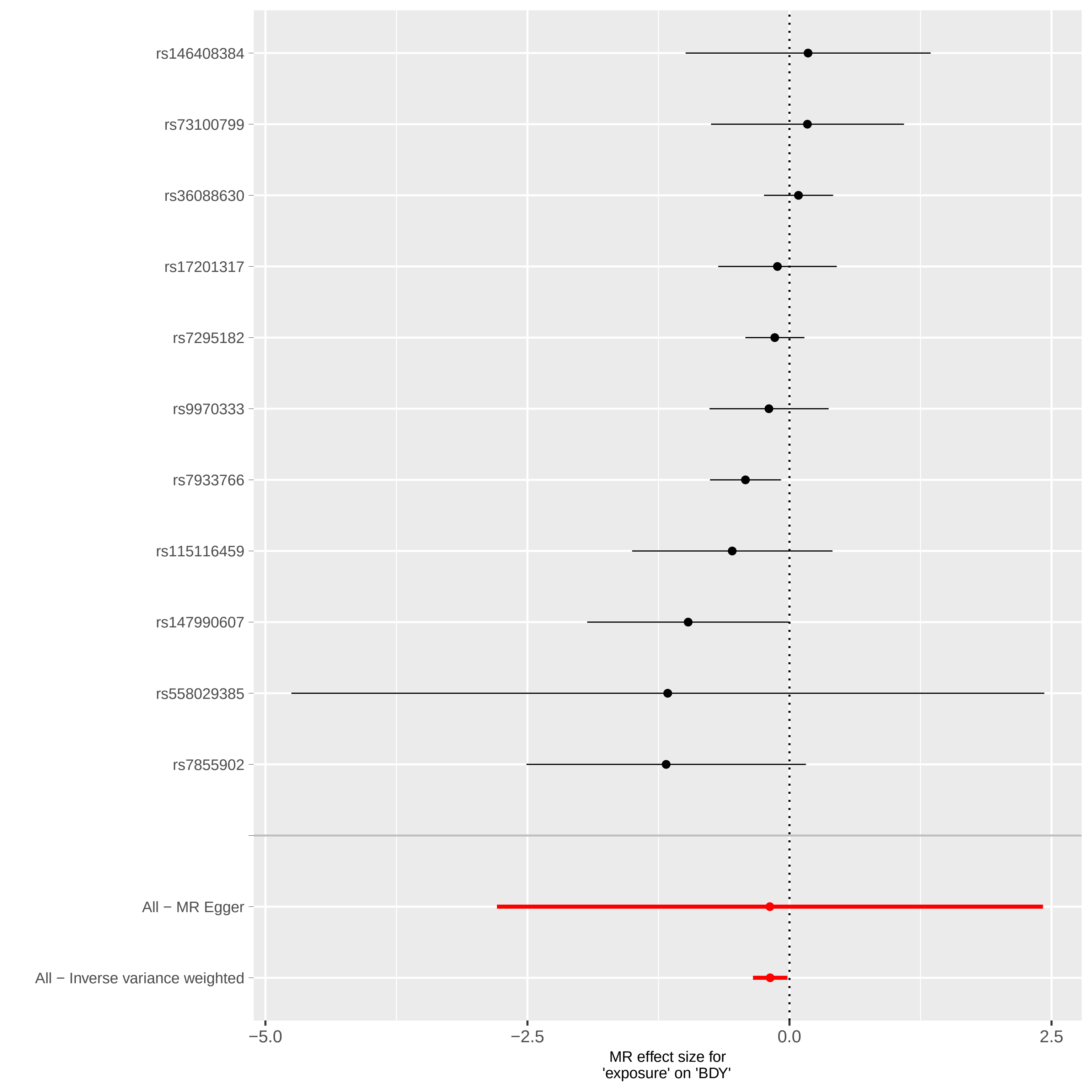

rs146408384
rs73100799
rs36088630
rs17201317
rs7295182
rs9970333
rs7933766
rs115116459
rs147990607
rs558029385
rs7855902
All − MR Egger
All − Inverse variance weighted
−5.0
−2.5
0.0
2.5
MR effect size for
'exposure' on 'BDY'
